# Environmental Impact of Flame Retardants (Persistence and Biodegradability)

**DOI:** 10.3390/ijerph6020478

**Published:** 2009-02-05

**Authors:** Osnat Segev, Ariel Kushmaro, Asher Brenner

**Affiliations:** 1 Unit of Environmental Engineering, Faculty of Engineering Sciences, Ben-Gurion University of the Negev, P.O.Box 653, Be’er-Sheva, 84105, Israel; E-Mails: segevos@bgu.ac.il (O. S.); brenner@bgu.ac.il (A. B.); 2 Department of Biotechnology Engineering, Faculty of Engineering Sciences, Ben-Gurion University of the Negev, P.O.Box 653, Be’er-Sheva, 84105, Israel

**Keywords:** Biodegradation, Bioremediation, Brominated flame retardants, Dehalogenation, Flame retardants, Persistence

## Abstract

Flame-retardants (FR) are a group of anthropogenic environmental contaminants used at relatively high concentrations in many applications. Currently, the largest market group of FRs is the brominated flame retardants (BFRs). Many of the BFRs are considered toxic, persistent and bioaccumulative. Bioremediation of contaminated water, soil and sediments is a possible solution for the problem. However, the main problem with this approach is the lack of knowledge concerning appropriate microorganisms, biochemical pathways and operational conditions facilitating degradation of these chemicals at an acceptable rate. This paper reviews and discusses current knowledge and recent developments related to the environmental fate and impact of FRs in natural systems and in engineered treatment processes.

## 1. Introduction

Flame retardants (FR) comprise a diverse group of chemicals which are widely used at relatively high concentrations in many applications, including the manufacture of electronic equipment, textiles, plastic polymers and in the car industry [1]. The annual consumption of flame retardants is currently over 1.5 million tonnes [2]. The use of FRs is primarily to protect materials against ignition and to prevent fire-related damage. Room combustion tests comparing FR plastics with non-FR plastics conducted by the National Bureau of Standards (The National Institute of Standards and Technology) have shown that FR materials allow longer escape time, less heat release, less smoke and release of a lower concentration of toxic gases. These effects are due to a decrease in the amount of burning materials [3].

**Figure 1.**
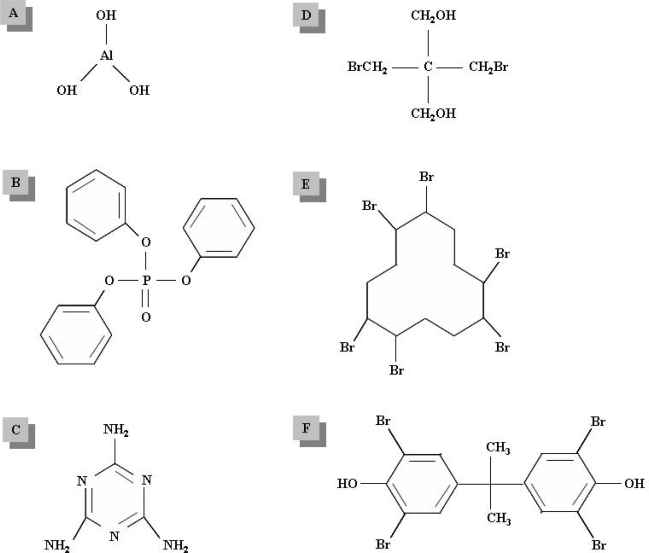
Chemical structures of different flame retardants (FR). (A) inorganic FR: aluminium hydroxide; (B) organophosphorus FR: triphenylphosphate (C) nitrogen-containing FR: melamine; (D–F) halogenated organic FRs: (D) aliphatic FR: dibromoneopentyl glycol (DBNPG) (E) cycloaliphatic FR: hexabromocyclododecane (HBCD) and (F) aromatic FR: tetrabromobisphenol A (TBBPA).

More than 175 different types of FRs exist, commonly divided into four major groups: inorganic FRs, organophosphorus FRs, nitrogen-containing FRs and halogenated organic FRs (Figure 1) [1,4]. Inorganic FRs comprise metal hydroxides (e.g. aluminium hydroxide and magnesium hydroxide), ammonium polyphosphate, boron salts, inorganic antimony, tin, zinc and molybdenum compounds, as well as elemental red phosphorous. Inorganic FRs are added as fillers into the polymers and are considered immobile, in contrast to the organic additive FRs. Organophosphorous FRs are primarily phosphate esters that may also contain bromine or chlorine. Organophosphorous FRs are widely used both in polymers and textile cellulose fibers. Nitrogen-containing FRs inhibit the formation of flammable gases and are primarily used in polymers containing nitrogen, such as polyurethane and polyamide. The most important nitrogen-based FRs are melamine and melamine derivatives. Halogenated organic FRs are usually based on chlorine and bromine. Brominated flame retardants (BFR) are more numerous than chlorinated FRs due to their efficiency and because at high temperatures, the decomposition products of brominated compounds are less volatile than are those derived from chlorinated compounds, since bromine is heavier than chlorine [5]. Based on their structure, halogenated FRs can be divided into three classes, namely aliphatic, cycloaliphatic and aromatic, as exemplified by dibromoneopentyl glycol (DBNPG), hexabromocyclododecane (HBCD) and tetrabromobisphenol A (TBBPA), respectively (Figure 1D–F) [4,6].

Although FRs chemically differ from one another, shared general mechanisms of action distinguish between various classes of FRs. Generally, FRs are divided as being gas-phase active or condensed-phase active, with the condensed-phase mechanisms of action being more common than the gas-phase ones. Further division is based upon the mechanism of action, namely chemical or physical. Some FRs (e.g. metal hydroxide like aluminum hydroxide) rely almost exclusively on a physical mechanism of action, whereas chemical mechanisms are always accompanied by one or more physical mechanisms. A combination of several mechanisms can be synergistic. The chemical mechanism of action of gas-phase-active FRs involves a scavenging of free radicals responsible for the branching of the radical chain reaction in the flame. The physical mechanism of action in the gas phase is to generate large amounts of non-combustible gases, which dilute flammable gases and decrease temperatures by absorbing heat. The most frequent condensed-phase mode of action is charring, which can be implemented by chemical interaction of the FR and the plastic polymer or by physical retention of the plastic polymer in the condensed phase [3].

The mode of incorporation into polymeric material divides the FRs into two additional classes, namely reactive or additive. Reactive FRs are chemically bonded into plastics, e.g. HET acid (chlorendic acid), TBBPA, DBNPG or different organophosphorus compounds. Additive FRs are numerous and more frequently used. They are blended with the polymers and are thus more likely to leach out of products, e.g. HBCD, aluminium trihydrate, magnesium hydroxide and phosphate esters [5,6].

FRs can find their way into the environment as wastewaters of industrial facilities that produce FRs and manufacturing facilities that incorporate such compounds into products, through volatilization and leaching from products during manufacturing or usage, upon breakdown of foam products or by disposal of products (e.g. electronic equipments), through leaching from landfills, combustion and recycling of waste products or adsorption onto dust particles [7,8]. In addition, additive FRs are believed to be more easily released into the environment than are reactive FRs [1]. Once a FR arrives into the environment, it can be attached to a particle for transport in water or delivery to the sediment or end up on an airborne dust particle and travel distances far from the production and/or emission site. Thus, traces of FRs (halogenated and organophosphorous-containing) are found in terrestrial, freshwater and marine ecosystems (i.e. in air, water, soil and sediments) at various locations, far from where they are produced and/or used [4,7,9–15].

Currently, because of their high performance efficiency and low cost, the largest market group of FRs is the BFRs [4]. Hence, we will further focus on the environmental impact of this group of compounds.

## 2. Brominated flame retardants

BFRs are used in a wide variety of indoor and outdoor products, including televisions, computers, microwave ovens, copy machines, lamp shades, textiles and furniture, where they constitute 5–30% of the product [4,6,8]. The global market demand for BFRs continues to grow and it is estimated that more than 200,000 tonnes of BFRs are produced globally each year. Among the many BFRs used world-wide, the main commercial BFRs are TBBPA, HBCD and polybromodiphenyl ethers (PBDE) [4]. All BFRs primarily act through chemical interference with the radical chain mechanism that takes place during the gas phase of combustion [16,17].

In general, halogen substituent characteristics, such as an electron-withdrawing ability and physical size and shape, impact the chemical reactivity of the compound. The physical size and shape of the halogen substituent may delay uptake into cells and enzymatic attack during biodegradation. The halogen moiety of an organic compound increases lipid solubility and reduces water solubility. In addition, the halogen substituent and its potential organohalide metabolites may increase the inherent toxicity of a compound. Thus, due to bromide(s) substituent(s), many BFRs are toxic (acute and chronic), persistent and bioaccumulate in the environment [18]. Since the early 1970s, increasing evidence as to the presence of different BFRs in the environment at various locations, far from where they are produced and/or used (the latest publications have even shown levels of BFRs in the Arctic) has amassed, eliciting enormous environmental concern. BFRs at different concentrations have been measured in indoor and outdoor air and dust samples [12,14,19–23], in water [24,25], in soil and sediment [12–14,22,26,27] and in sewage sludge [13,22,26,28]. BFRs are detected in plants and wildlife throughout the food chain, in human tissues, blood serum and breast milk of exposed occupational populations (i.e. individuals working in the production of BFRs or production, recycling, or disposal of BFR-containing products) and in the general populations [12–14,20–22,25,26,29–42]. Furthermore, toxic (acute and chronic) and ecotoxic effects of some BFRs have been observed, including immunotoxicity, cytotoxicity, neurotoxicity, endocrine disruption, genotoxicity, mutagenicity, carcinogenicity and teratogenicity [4,6,8,26,27,43–47]. Despite these observations, only limited information is available on many BFRs, especially concerning the effect of BFRs on wildlife and man, their environmental fates and biodegradability potential.

## 3. Environmental Fate of Brominated Flame Retardants

Like most of the halogenated organic compounds, BFRs generally have limited biodegradability, are persistent and tend to accumulate in the environment. However, in certain environmental conditions, a number of abiotic and biotic processes can occur. Abiotic processes are physical-chemical processes that include photodegradation, wet and dry deposition, decomposition at high temperature, chemical reactions with other compounds or radicals (e.g. hydroxyl, metals, etc.) that are present in the environment and changes in compound characteristics due to environmental factors, like temperature and pH. Biotic processes can be defined as those biological processes that include bioaccumulation and entry into the food chain, biotransformation and biodegradation. All of these processes have the most significant environmental relevance when discussing the environmental fate of BFRs, as well as remediation of contaminated sites and risk assessment since such situations can change compound properties, including mobility, bioavailability and toxicity. Consequently, a compound can be modified to be less or even more toxic to plants, wildlife and man than was the original compound.

Photodegradation is a physical process known to occur naturally in the environment. Different PBDE congeners, like decabrominated diphenyl ether (BDE-209), can be photochemically degraded (via either UV or sunlight) in organic and aqueous solvent systems and in soils and sediments. Degradation results in the formation of less brominated PBDEs that can form more persistent and toxic lower brominated compounds [48,49]. Raff *et al.* estimated that 90% of lower brominated PBDEs, like 2,2′,4,4′-tetrabromodiphenyl ether (BDE-47), that enter the lower troposphere may be removed by photolysis before being deposited, with wet and dry deposition accounting for more then 95% of decabromodiphenyl ether (BDE-209) removal. Moreover, deposition processes control the loss of BDE-209 from the atmosphere and are responsible for the enhancement of BDE-209 found in sediment samples taken from lakes and rivers around the world [50]. TBBPA is also photolytically decomposed when exposed to UV light. The breakdown products correspond to different brominated organic compounds, like dibromophenol, 2,4,6-tribromophenol and di- and tribromobisphenol A [26].

There are studies that indicate that during thermal stress, PBDEs and TBBPA are converted to the dioxin-like compounds, polybrominated dibenzodioxins (PBDDs) and polybrominated dibenzofurans (PBDFs) [8].

Another process that may alter the bioavailability of compounds is changes in compound solubility due to environmental factors. For example, at neutral pH, TBBPA has very low solubility and its soil mobility is expected to be minimal. However, at higher pH (as in some arid soils areas), the solubility of TBBPA increases, making its soil mobility and potential for groundwater contamination considerable [51].

An important biotic process that affects the environmental ecotoxicity potential of a compound is its bioaccumulation. There is increasing evidence that some BFRs, like PBDE and HBCD, bioaccumulate in the food chain, as increasing concentrations of these BFRs are found in species higher in the food chain, such as zooplankton, invertebrates, fish and sea mammals [14,52–54]. Bioaccumulation of BFRs in the food chain is one of the ways that human can be exposed to BFRs, namely through diet, upon eating contaminated fish, meat, eggs, dairy products, *etc* [55].

### 3.1. Biodegradation and Bioremediation

Biodegradation process is one of the most significant processes experienced by organic compounds in the environment. The biodegradation process, which is (largely) mediated by microorganisms, can contribute to the reduction/complete elimination of organic contaminants in the environment [18]. Ideally, biodegradation of compounds in the environment would result in complete mineralization. However, this is not always the case, especially with complex compounds [56,57]. Aerobic and anaerobic biodegradation processes of halogenated organic compounds other than BFRs have been frequently reported [18,58–60]. Microorganisms can use halogenated organic compounds in four ways, namely as a carbon source and oxidizable substrate, as an electron acceptor in the ‘halorespiration’ process, in co-metabolic transformation, and in fermentative metabolism, in which a dehalogenated intermediate serves as an electron acceptor [61]. Halogen removal, i.e. the dehalogenation step, is the key reaction during the biodegradation of halogenated organic compounds. A number of dehalogenation enzymatic mechanisms are known to occur aerobically and anaerobically, mediated by a wide range of microorganisms [59–62]. Usually, halogen removal reduces compound resistance to biodegradation and the risk of forming toxic intermediates during subsequent metabolic steps [61]. However, this is not always the case, especially if the transformation does not result in complete mineralization of the compound such that brominated substituents remain.

**Figure 2.**
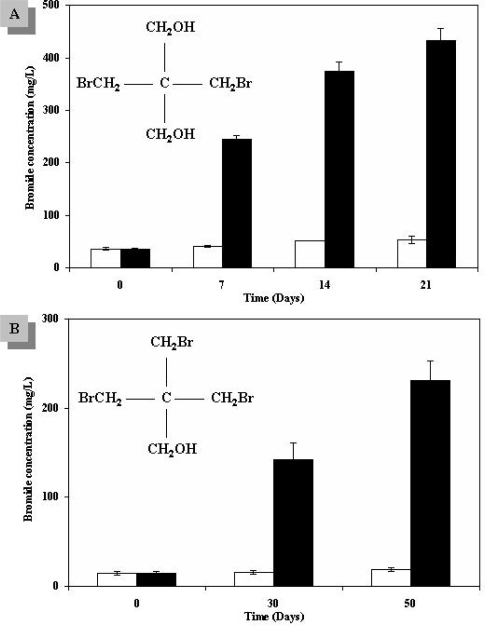
Debromination of dibromoneopentyl glycol (DBNPG) and tribromoneopentyl alcohol (TBNPA) by a bacterial consortium. (A) Bromide concentration in a DBNPG enrichment culture. (B) Bromide concentration in a TBNPA enrichment culture. The increase of bromide concentration in both culture is shown (black), as opposed to the value in control cultures (white) [63,64].

**Figure 3.**
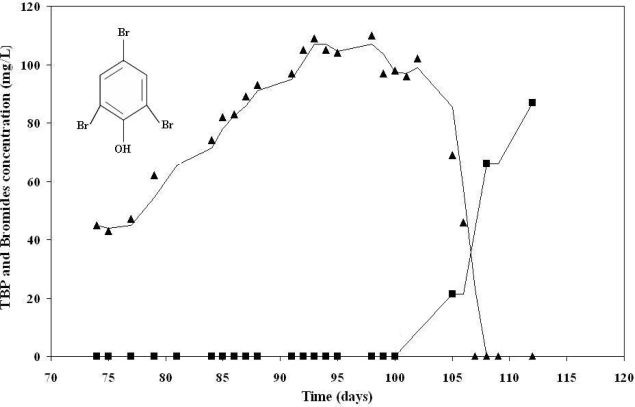
2,4,6 Tribromophenol (TBP) aerobic biodegradation by aerobic cultures simulating activated sludge processes. TBP concentration (▴) in the reactor decreases while the bromide concentration (▪) increases. The accumulation of the bromide indicates a debromination reaction [66].

Recently, several studies have been conducted addressing the biodegradation of different BFRs. Segev *et al.* have demonstrated the biodegradation of two aliphatic BFRs, dibromoneopentyl glycol (DBNPG) and tribromoneopentyl alcohol (TBNPA), under aerobic conditions by a bacterial consortium originating from soil sediments from a contaminated site. The biodegradation was accompanied by a release of bromide into the medium by a bacterial debromination reaction (Figure 2). Among the consortium members are known dehalogenating bacterial species [63,64]. 2,4,6-tribromophenol (TBP) was found to be aerobically biodegraded during transport in low permeability fractured chalk cores. During transport, the physicochemical condition of the medium (e.g. an aquifer) must be considered, in addition to biological factors, such as residence time and physicochemical characteristics of the rock [65]. Brenner *et al.* tested various reactor systems added with contaminated sediments and cultivated with concentrated biomass acclimated to the treatment of industrial wastes for the biological treatment of a waste mixture containing bromo-organic compounds, including TBBPA and TBP. Although various redox conditions were applied and different carbon sources were tested, no TBBPA biodegradation was observed. TBP, on the other hand, was found to be easily biodegraded by aerobic cultures simulating the activated sludge process (Figure 3). This was linked to a consistent accumulation of bromides, released to the liquid following TBP breakdown [66]. TBP has also been found to undergo anaerobic biodegradation by different bacteria, such as *Achromobacter piechaudii*, *Desulfovibrio* stain TBP-1 and *Ochrobacterium* sp. strain TB01. The primary step in TBP biodegradation is an anaerobic reductive debromination reaction in which the halogenated organic compound is used as a final electron acceptor in a ‘halorespiration’ process [67–69]. Recent publications have demonstrated the anaerobic reductive debromination of different PBDEs, including decabromodiphenyl ether (BDE-209), by isolated bacteria, mixed cultures and fungi in mineral medium, as well as by sediment or sewage sludge [48,70–74]. The product generated from BDE-209 after debromination is more toxic and bioaccumulative than is the starting compound [72]. Davis *et al.* (2005) have shown HBCD biodegradation in aerobic and anaerobic soil and aquatic sediments. In this process, HBCD is sequentially debrominated by a dehalogenation mechanism known as dihaloelimination, where, at each step, there is a loss of two bromines from vicinal carbons with subsequent formation of a double bond between neighboring carbon atoms [75,76]. Ronen *et al.* described reductive debromination of TBBPA to bisphenol-A (BPA), a known estrogenic compound and suspected teratogen, under anaerobic conditions [77,78]. BPA can then undergo aerobic mineralization mediated by the Gram-negative aerobic bacterium strain WH1 of the genus *Sphingomonas* [57].

A compound can undergo other biotransformation reactions beside biodegradation in the environment. The origin of a dimethylated derivative of TBBPA that was found in sediments may be the methylation of TBBPA by microorganisms [26]. Indeed, Allard *et al.* have shown that *Rhodococcus* sp. strain 1395 is capable of O-methylating TBBPA under aerobic conditions. The reaction product is highly lipophilic, making the derivative more bioaccumulable than is TBBPA [79].

Remediation of contaminated sites by physical-chemical processes is expensive and labor-intensive and often results in shuffling toxicants from one place to another. A possible solution to this problem is intensive, targeted biological treatment based on the biodegradation of a compound by microorganisms. The fact that a compound in aqueous solution is utilized by microorganisms does not guarantee that the compound will be biodegradaed by the same microorganism in a complex environmental matrix, like soil, aquifer or sediment, all often associated with natural organic matter [18]. There are few studies regarding the bioremediation of halogenated organic compounds in contaminated sites [80]. It is thus essential to expand our knowledge and develop tools that will allow better understanding and mimicking of natural environmental conditions so as to enhance bioremediation of BFR-contaminated sites.

## 4. Summary

FRs constitute a large and diverse group of anthropogenic environmental contaminants used in numerous applications, including the manufacture of electronic equipment, textiles, plastic polymers and in the car industry, primarily to protect materials against ignition. Among the different FRs groups, the BFRs are the largest market group, because of their high performance efficiency and low cost. Recent reports have demonstrated the presence of BFRs at various concentrations in air, water, soil, wastewater and sediments far from the locations where they are produced. Moreover, traces of BFRs have been found in plants, wildlife and even in human samples. Thus, recent publications on the persistence, bioaccumulation and toxicity of some of BFRs are a source of great concern. Despite the information available on BFR distribution, many questions related to exposure, toxicology, metabolism and environmental occurrence and behavior remain unanswered. Remediation of contaminated sites by physical-chemical processes is expensive and labor-intensive and often results in shuffling toxicants from one place to another. A possible solution to this problem is thus controlled, targeted biological treatment. Taking into account the limited information available today, more research is needed regarding the fate and impact of FRs in the environment. Since certain abiotic and biotic processes can change compound characteristics and make them more persistent and toxic, it is important to investigate and understand the detailed biochemical transformation and degradation pathways, the microorganisms involved and the potential derivatives and intermediates generated.
